# Loeffler endocarditis with intracardiac thrombus: case report and literature review

**DOI:** 10.1186/s12872-021-02443-2

**Published:** 2021-12-28

**Authors:** Qian Zhang, Daoyuan Si, Zhongfan Zhang, Wenqi Zhang

**Affiliations:** Department of Cardiology, China-Japan Union Hospital of Jilin University, Xiantai Street No. 126, Changchun, Jilin China

**Keywords:** Case report, Eosinophilia, Hypereosinophilic syndrome, Intracardiac thrombus, Loeffler endocarditis

## Abstract

**Background:**

Loeffler endocarditis is a relatively rare and potentially life-threatening heart disease. This study aimed to identify the characteristic features of Loeffler endocarditis with intracardiac thrombus on a background of hypereosinophilic syndrome (HES).

**Case presentation:**

We described a 57-year-old woman with Loeffler endocarditis and intracardiac thrombus initially presenting with neurological symptoms, who had an embolic stroke in the setting of HES. After cardiac magnetic resonance (CMR), corticosteroids and warfarin were administered to control eosinophilia and thrombi, respectively. During a 10-month follow-up, the patient performed relatively well, with no adverse events. We also systematically searched PubMed and Embase for cases of Loeffler endocarditis with intracardiac thrombus published until July 2021. A total of 32 studies were eligible and included in our analysis. Further, 36.4% of recruited patients developed thromboembolic complications, and the mortality rate was relatively high (27.3%). CMR was a powerful noninvasive modality in providing diagnostic and follow-up information in these patients. Steroids were administered in 81.8% of patients, achieving a rapid decrease in the eosinophil count. Also, 69.7% of patients were treated with anticoagulant therapy, and the thrombus was completely resolved in 42.4% of patients. Heart failure and patients not treated with anticoagulation were associated with poor outcomes.

**Conclusions:**

Cardiac involvement in HES, especially Loeffler endocarditis with intracardiac thrombus, carries a pessimistic prognosis and significant mortality. Early steroids and anticoagulation therapy may be beneficial once a working diagnosis is established. Further studies are needed to provide evidence-based evidence for managing this uncommon manifestation of HES.

## Background

Hypereosinophilic syndrome (HES) is a rare disorder characterized by the elevation of blood eosinophil count (> 1.5 × 10^9^/L) and multiple-organ involvement directly attributable to eosinophilia [1]. Loeffler endocarditis involves the abnormal infiltration of eosinophils into the endomyocardium, with subsequent tissue damage and fibrosis resulting from eosinophil degranulation, eventually leading to impaired diastolic function and restrictive ventricular filling [2]. It is divided into three pathological stages: necrotic stage, thrombotic stage, and fibrotic stage [3]. Notably, systemic thromboembolic events after mural thrombus formation and cardiac manifestations are considered to be common causes of morbidity and mortality in HES [4]. Considering the less recognized and high in-hospital mortality of patients with Loeffler endocarditis, data regarding their clinical presentations, courses, and outcomes remain uncertain. Furthermore, some evidence supports the effectiveness of steroids [5]. However, the guidelines and consensus statements regarding the treatment of Loeffler endocarditis are not clear. Therefore, this study aimed to present an unusual case of Loeffler endocarditis and intracardiac thrombus that caused cerebral embolic infarctions, and to conduct a systematic review on published cases of Loeffler endocarditis with intracardiac thrombus, summarizing clinical manifestations, diagnosis, treatments, and outcomes.

## Case presentation

**Fig. 1:**
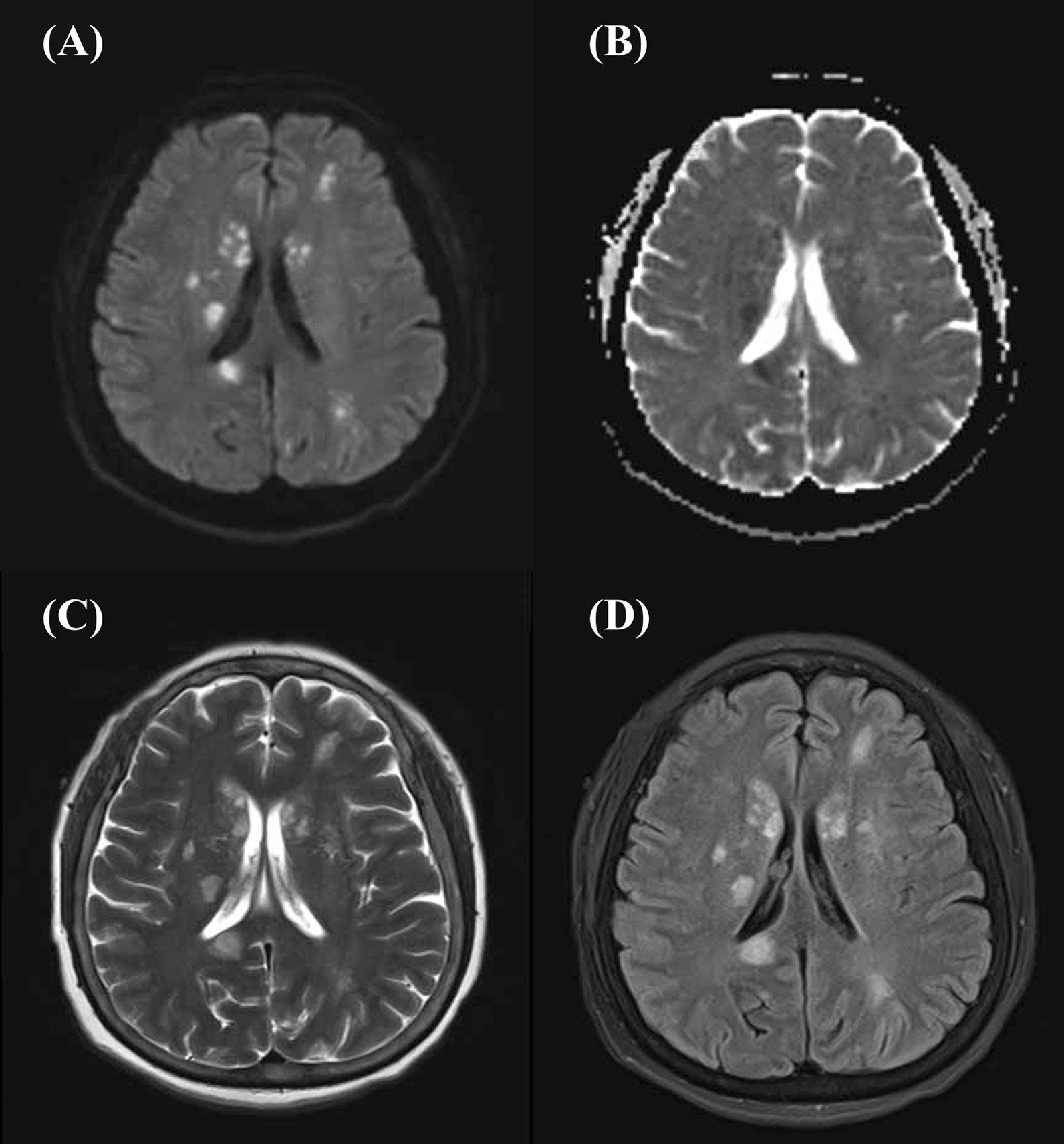
Brain magnetic resonance imaging. **A** Diffuse-weighted image (DWI), **B** apparent diffusion coefficient (ADC) map, **C** T2-weighted image, and **D** fluid attenuated inversion recovery (FLAIR) image. Multifocal lesions of high intensity on DWI and ADC maps showed low values. There were multiple T2 high signal lesions in bilateral cerebral white matter

**Fig. 2:**
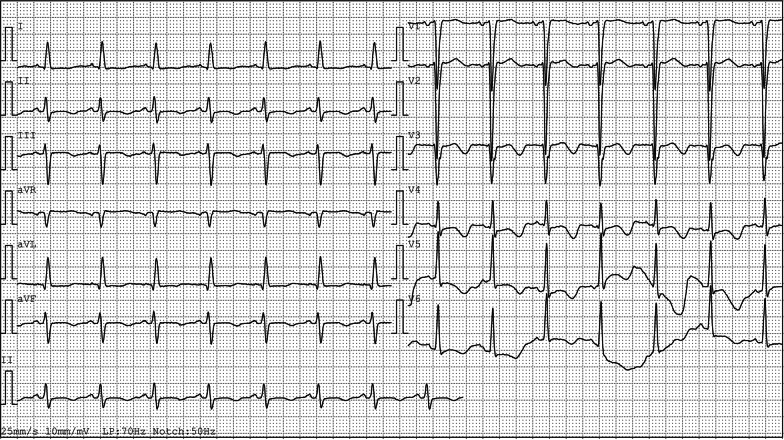
Electrocardiogram: normal sinus rhythm, T wave inversion in leads II, III, aVF, V3,V4, V5, V6

**Fig. 3:**
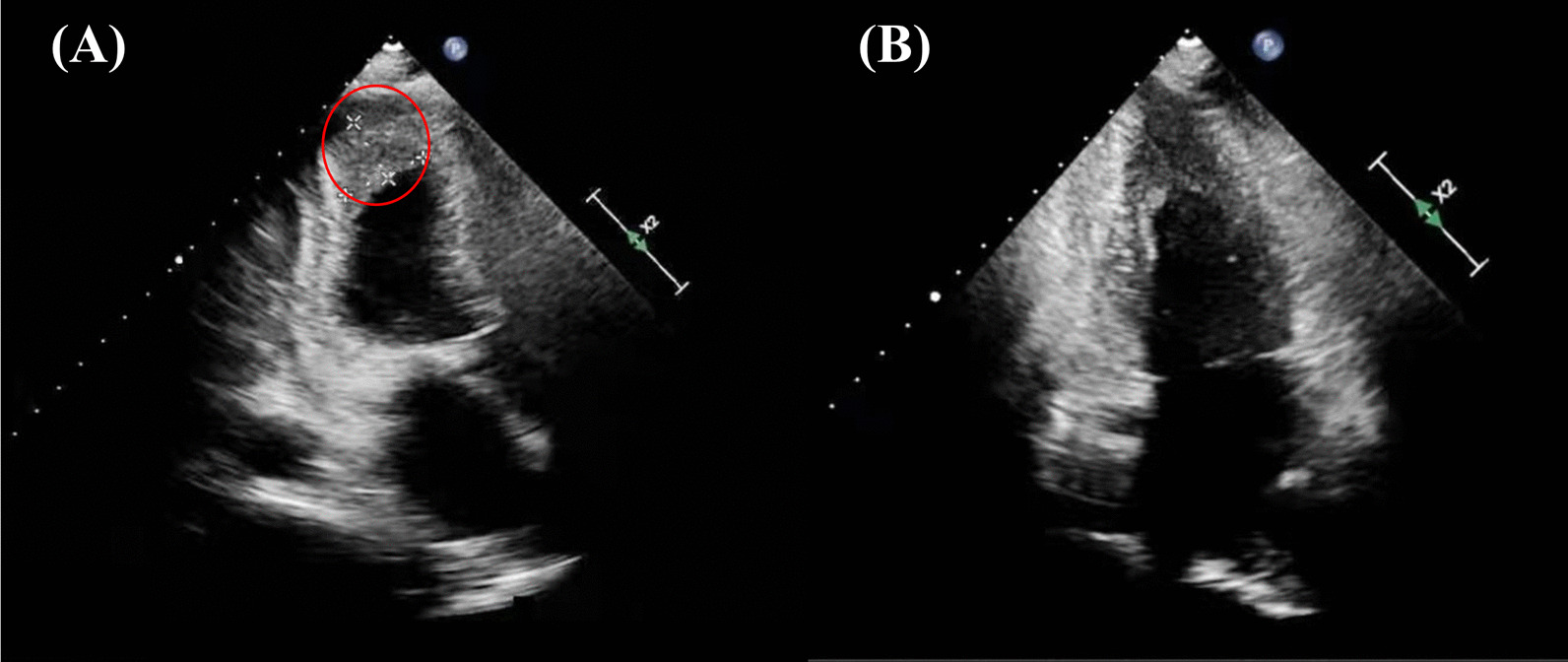
**A** Apical four-chamber view of the transthoracic echocardiogram showing thickened left ventricular endocardium and left ventricular thrombus formation (red circle). **B** Ten-month follow-up echocardiographic imaging after treatment showing thickening of the left ventricular apex and suspicion of apical thrombus

**Fig. 4:**
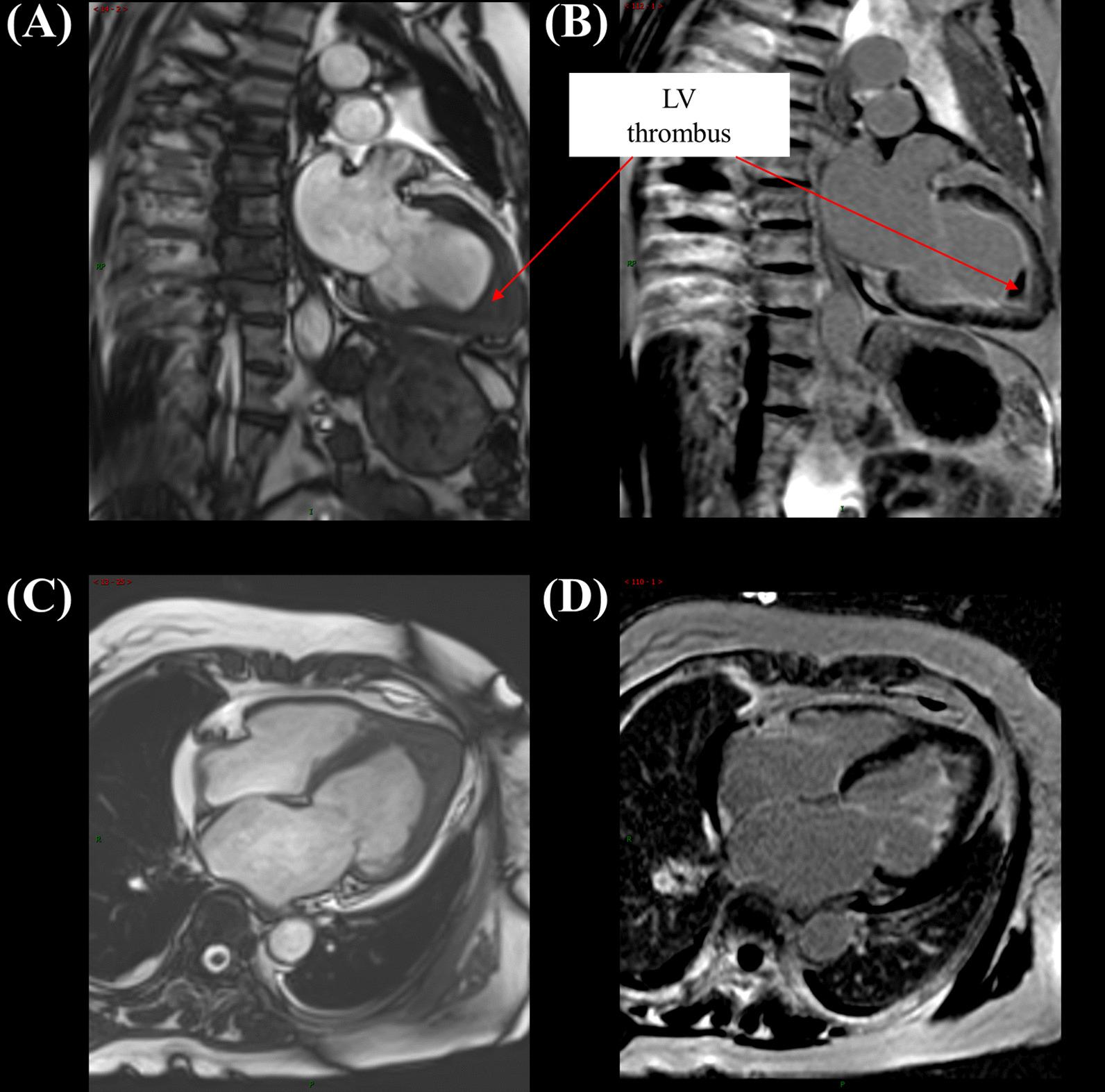
**A**, **C** Early gadolinium enhancement imaging demonstrating a hypointense filling defect at the left ventricular apex. **B**, **D** Late gadolinium enhancement imaging demonstrating a large left ventricular apical thrombus and hyperenhancement indicative of endomyocardial fibrosis

A 57-year-old woman with a known history of asthma and rheumatoid arthritis was admitted to the hospital after presenting with a headache and dyspnea for 1 week. The neurological examination showed vague speech, mild dysarthria, and limb muscle strength level 3. Her brain magnetic resonance imaging showed multifocal acute-to-subacute ischemic lesions widely distributed over the bilateral cerebellar hemispheres and thalamus and parenchymal hemorrhage (Fig. 1). The initial laboratory findings were as follows: the white blood cell count was 20.43 × 10^9^ (normal range, 4–10 × 10^9^/L), with increased peripheral eosinophilia at 12.04 × 10^9^/L (normal range, 0.05–0.50 × 10^9^/L). Other inflammatory parameters showed an increased erythrocyte sedimentation rate of 79 mm/h (normal range, 0–20 mm/h) and a C-reactive protein level of 95.4 mg/L (normal range, 0–8 mg/L). The patient was also positive for the anti-antineutrophilic perinuclear antibody (pANCA). Other specific markers related to autoimmune diseases, such as anti-dsDNA and anti-Sm, rheumatoid factorare were all negative. Her travel history was unremarkable. Her hospital course was further complicated by severe shortness of breath and elevated cardiac enzyme levels (cTnI: 14.10 ng/mL, CK-MB: 41.9 U/L, NT-proBNP: 28,700 ng/mL), which prompted an extensive cardiac workup. The electrocardiogram showed T-wave inversions in leads II, III, and aVF (Fig. 2). Two-dimensional echocardiography (Fig. 3A) showed that the systolic function of the heart was within the normal range, which was found to be 59% by the Simpson method. A thickened left ventricular (LV) endocardium with markedly solid echo could be seen, and a diagnosis of LV thrombus formation was suggested. Severe mitral regurgitation and moderate tricuspid regurgitation were noted. A small amount of pericardial fluid was also present (4.6 mm). We further excluded other causes of eosinophilia, such as malignancy, autoimmune diseases, and drug reactions. The parasites were negative in stool culture. In the peripheral blood smear, most of the granulocytes were normal, with no clonal proliferation or primordial cells. Further diagnostic clarification was required. The cardiac magnetic resonance (CMR) confirmed the presence of a thrombus measuring approximately 1.5 × 1.7 cm in the apex of the LV, which, we believed, was the source of cerebral embolization. Gadolinium-enhanced CMR showed striated delayed enhancement between the apex and the papillary muscles restricted to the endocardium, with decreased diastolic function (Fig. 4), which was consistent with extensive endomyocardial fibrosis. Endocardial biopsy was recommended as the diagnostic gold standard to verify the histopathologic features. However, considering the risk of this invasive operation, the patient refused.

**Fig. 5:**
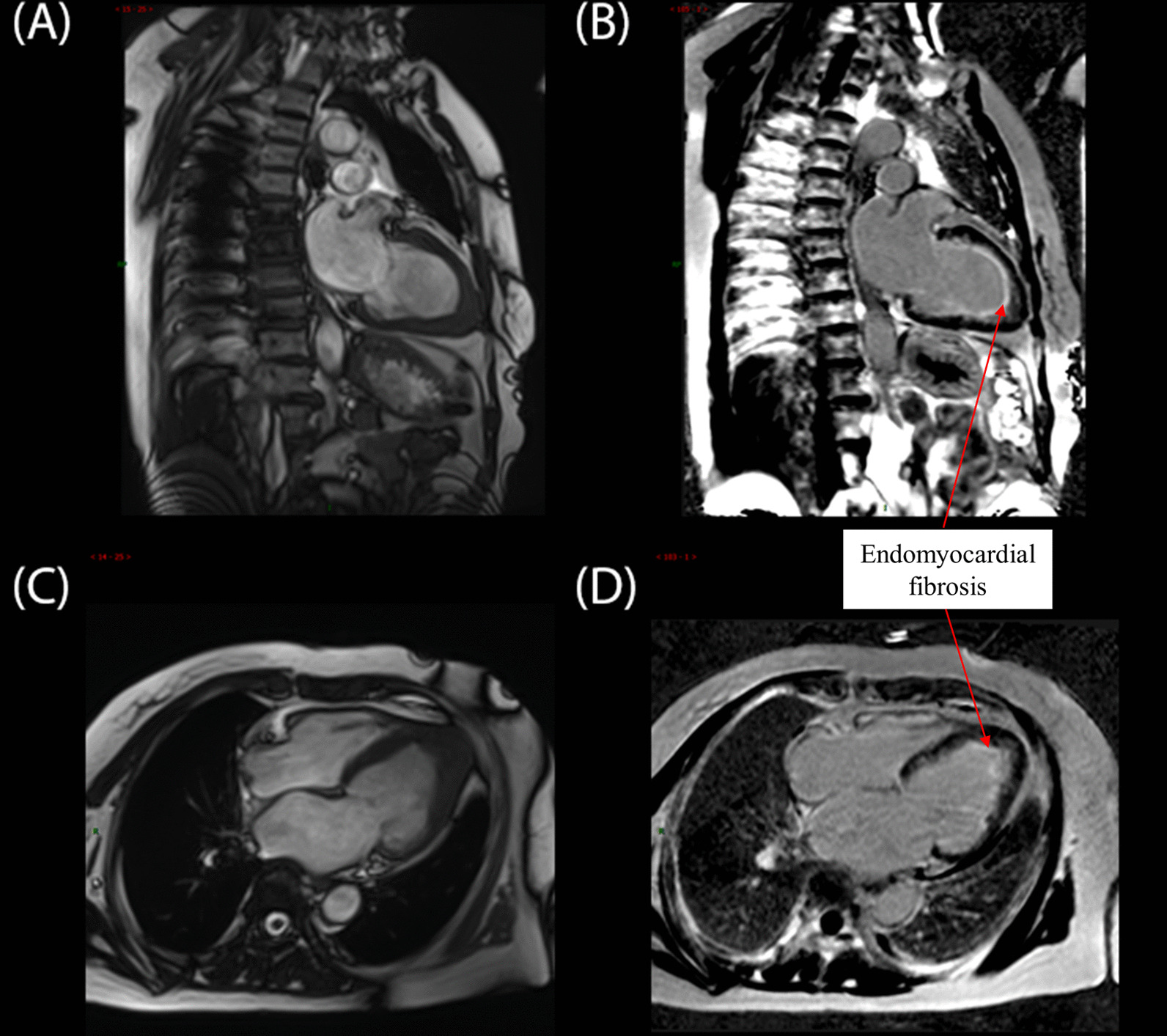
At the 10-month follow-up, early and late gadolinium enhancement imaging demonstrating left ventricular apical thrombus resolution and endomyocardial fibrosis still existing after treatment

Based on the diagnosis of Loeffler endocarditis with LV thrombus, an intravenous bolus of a corticosteroid [prednisolone 1 mg/(kg·day)] was initiated, followed by 40 mg per day orally, which was prescribed as a definitive line of therapy. The blood tests showed significantly decreased eosinophil counts and percentages after another 2 days, along with a normalized cTnT level. The patient was treated with rivaroxaban to dissolve the thrombus, but the thrombus did not shrink after 3 months. Then, warfarin was added as antithrombotic therapy until follow-up. She was doing relatively well, and no adverse events occurred during a 10-month follow-up period. The echocardiography showed apical hypertrophy, and a suspected thrombus still existed (Fig. 3B). Therefore, CMR (Fig. 5) was repeated, which revealed the complete resolution of the apical thrombus and apical hypertrophy. However, no clear regression of endomyocardial fibrosis was observed. The timeline table is shown in Table 1.

**Table 1:** Time line table from presentation to the last follow up

	In hospital	5 days after treatment	4-month follow-up	10-month follow-up
*Laboratory findings*				
WBC (10^9^/L)	20.43	11.94	5.5	7.85
Eosinophilia (10^9^/L)	12.04	6.53	0.28	0.08
cTnI (ng/mL)	14.10	0.35	0.01	NA
NT-proBNP (ng/mL)	28,700	22,300	3230	NA
ESR (mm/h)	79	NA	NA	NA
CRP (mg/L)	95.4	NA	NA	NA
*Echocardiography*				
LVEF, %	59	64.3	58	60
Thrombus size (mm)	16.5*16.4	24*18	23*9	NA
*Treatments*				
Steroid	1 mg/kg/day (intravenous bolus)	40 mg	NA	NA
Antithrombotic	rivaroxaban	Warfarin	Warfarin	NA

WBC, White blood cell; ESR, erythrocyte sedimentation rate; CRP, c-reactive protein; LVEF, left ventricular ejection fraction; NA, not available

## Literature search strategy

For the literature review, we searched the PubMed and Embase for relevant studies, including all case reports and case series, published until June 1, 2021. The database was created using the search phrases “Loeffler endocarditis,” “hypereosinophilic syndrome,” and “thrombus.” Studies that described Loeffler endocarditis related to thrombus formation were selected. Patients with specific diseases, including tropical endomyocardial fibrosis, Churg-Strauss syndrome, eosinophilic pneumonia, and clear heart disease combined with thrombus, were excluded. Cases were selected only if sufficient data were available for each case series. Two authors extracted and verified the data independently, and any differences were resolved through discussion. A total of 33 cases were identified. We also used the reference lists of articles published in English for the manual search. The clinical characteristics, complete blood counts, echocardiograms, CMR, treatment monitoring, and clinical follow-up were reviewed.

## Results

We initially identified 477 articles using the aforementioned search strategy. A total of 33 [6–37] cases of Loeffler endocarditis associated with endoventricular thrombus were found. The epidemiological data, clinical manifestations, diagnostics, treatments, and outcomes are summarized in Table 2. The incidence of embolic stroke was 36.4% among patients with thrombi. The median age of patients was 44 years (IQR: 26–60 years), and the male-to-female ratio was 13:20. At admission, the most common presenting complaint was dyspnea (63.64%), followed by fever (30.30%), nervous system symptoms (18.18%), chest pain (15.15%), fatigue (15.15%), abdominal symptoms (12.12%), cough (6.06%), and palpitations (6.06%). At presentation, 82.0% of cases presented with an unremarkable electrocardiogram, and 24.24% of cases had increased troponin levels. Subsequently, common echocardiographic findings included mitral regurgitation (42.42%) and aortic regurgitation (4%); 15% of patients had the involvement of two valves. A large number of cardiac structures were affected in these patients; however, the ejection fraction was well maintained. Pericardial effusions were observed in 18.18% of patients. Myocardial fibrosis and endoventricular thrombus were usually detected using delayed-enhancement gadolinium imaging. In terms of management, steroid therapy was the most common therapeutic modality (81.8%), and immunosuppression was added in three cases (10%).

**Table 2:** Clinical summary of the 33 cases of loeffler endocarditis with intracardiac thrombus

Study	Sex	Age	Clinical presentation	Eosinophil proportion	Increased troponin I	Valvulopathy/pericardial fluid	Treatment	Surgical intervention	Diagnostic methods	Cardiac dysfunction	Stroke	Evidence of thrombus	Prognosis
Lin et al. [6]	M	59	Dyspnea	NA	Yes	AR + MR/No	Corticosteroid + Immunosuppression + Warfarin	No	Endomyoca-rdial biopsy + TTE	Yes	No	L	Thrombus Regression + Doing well
Hwang et al. [7]	M	55	Dyspnea, left-sided weakness	54.9%	Yes	No/ No	Corticosteroid + Hydroxyurea + Warfarin	No	TTE	No	Yes	L	Thrombus regression
Demetriades et al. [8]	F	57	Headache, lethargy, and reduced consciousness	59.1%	NA	No / No	Corticosteroid + Warfarin	No	CMR	No	Yes	L	Thrombus regression Doing well
Afzal et al. [9]	F	66	Dyspnea	1.13 k/microL	NA	MR/ No	Corticosteroid + Warfarin	No	CMR	No	No	L	Thrombus regression
Morgan et al. [10]	M	35	Dyspnea	4.5%	Yes	No / Yes	Corticosteroid + Warfarin	No	CMR	No	No	L	Thrombus regression
Kumar et al. [11]	M	14	Fever, cough, chest pain	50.3%	NA	No / No	Corticosteroid	No	CMR	No	No	L	Thrombus NA Doing well
Kalra et al. [12]	M	61	Dyspnea	55.0%	NA	MR	Corticosteroid + Immunosuppres-sion	No	CMR	Yes	No	L	Died from bacterial sepsis
Dufour et al. [13]	F	16	Fever, chest pain	NA	Yes	NA	Corticosteroid	No	CMR	No	No	L	Thrombus regression
Kim et al. [14]	M	28	Headache, dyspnea	46.6%	NA	NA	Immunosuppres-sion + imatinib + anticoagulation	No	Endomyoca-rdial biopsy	No	Yes	L + R	Thrombus regression
Massin et al. [15]	M	12	Fever, dyspnea	71.0%	NA	MR	Corticosteroid + Warfarin	Endomyocard-ectomy	CMR	Yes	No	L	Death(septic shock)
Ammirati et al. [16]	M	65	Palpitations, dyspnea	18.0%	N	MR + TR	Corticosteroid + anticoagulation	No	CMR + Endomyoca-rdial biopsy	Yes	Yes	L	Doing well
Casavecchia et al. [17]	F	44	Fever, dyspnea	35.7%	NA	MR + TR/ Y	Corticosteroid + Immunosuppres-sion + Warfarin	No	CMR	Yes	No	L + R	Presence of thrombus
Wright et al. [18]	F	46	Fever, dyspnea	85.0%	NA	MR	Corticosteroid + interferon alfa	Valve replacement	Endomyoca-rdial biopsy + TTE	Yes	No	L	Thrombus NA Poor prognosis
Toshimitsu et al. [19]	M	57	Numbness of the lower extremities	55.0%	NA	No / No	Corticosteroid + Warfarin	No	TTE	No	No	L	Shrunk thrombus
Tai et al. [20]	F	4	Fever, dyspnea	83.0%	NA	No/ No	Corticosteroid + Warfarin	No	CMR	No	No	R	Thrombus regression
Saito et al. [21]	F	59	Fever	30.0%	Yes	No/ Yes	Corticosteroid + Immunosuppres-sion + Warfarin	No	TTE + CMR	NA	No	L	Thrombus regression
Gupta et al. [22]	F	17	Fever, dyspnea	30.0%	NA	MR + TR	Corticosteroid + Warfarin	No	TTE	NA	No	L + R	Presence of thrombus
Kharabish et al. [23]	F	36	Dyspnea	28.0%	NA	MR/ Yes	Corticosteroid + + Warfarin	No	CMR	NA	No	L	Thrombus regression
Van et al. [24]	F	51	Dyspnea, chest pain	46.0%	Yes	MR/ No	Corticosteroid + Immunosuppres-sion + Warfarin	No	CMR	Yes	No	L	Thrombus Regression
Lee et al. [25]	M	60	Dyspnea	53.0%	NA	No/ No	Corticosteroid + Warfarin	No	TTE	Yes	No	L	Presence of thrombus
Thaden et al. [26]	F	40	Abdominal pain, diarrhea	NA	Yes	MR/ No	Warfarin	Surgical excision of thrombus and Valve replacement	Endomyocardial biopsy	Yes	No	L + R	Surgical excision of thrombus Poor prognosis
Koneru et al. [27]	F	24	Nervous system symptoms	49.1%	NA	No/ No	Corticosteroid + Immunosuppres-sion + Warfarin	No	CMR	NA	Yes	L	Thrombus regression
Francone et al. [28]	M	12	Fever, dyspnea	41.4%	NA	No/ No	Corticosteroid + Warfarin	No	CMR	Yes	Yes	L	Died from cerebral stroke
Coelho et al. [29]	M	56	Palpitations, dyspnea, chest pain	47.0%	Yes	No/ No	Corticosteroid	No	CMR	NA	Yes	R	NA
Chad et al. [30]	F	71	Dyspnea	24.0%	NA	MR + TR	Corticosteroid + Warfarin	No	CMR	Yes	No	L	Death (Heart failure)
Amini et al. [31]	F	74	Chest pain	64.0%	Yes	MR/ Yes	Corticosteroid	No	Endomyoca-rdial biopsy	Yes	Yes	R	Thrombus NA Poor prognosis
Chang et al. [32]	F	35	Left-side weakness	NA	NA	No/ No	Corticosteroid + Warfarin	No	CMR	N A	Yes	L	Improved
Lin CH et al. [33]	F	67	Dyspnea, chest pain	NA	NA	NA	Corticosteroid + Warfarin	No	CMR	No	Yes	L	NA
Tanaka et al. [34]	F	65	Dyspnea	NA	NA	MR/ No	NA	No	TTE	Yes	Yes	L	Died from stroke
Ucxar et al. Case 1 [35]	F	35	Abdominal distention	13.0%	NA	No/ No	Corticosteroid	No	TTE	Yes	No	L	Died from refractory heart failure
Case 2 [35]	F	53	Fever, abdominal pain, dyspnea, cough	15.0%	NA	MR + TR/ No	Corticosteroid + Warfarin	No	TTE	N A	No	L + R	Death
Salanitri et al. [36]	F	59	Fatigue, Abdominal pain	10.0%	NA	No/Yes	Corticosteroid + Hydroxyurea	No	Endomyoca-rdial biopsy	N A	No	L + R	Died from septicemia
Kocaturk et al. yyy [37]	M	17	Fever, dyspnea	45.4%	NA	No/ No	Corticosteroid + Hydroxyurea	No	TTE	N A	Yes	L	Died from stroke

TTE, transthoracic echocardiography; MR, mitral regurgitation; TR, tricuspid regurgitation; AR, aortic regurgitation; L, In the left ventricle; R, In the right ventricle; CVA, cerebrovascular accident; NA, not available

In most patients, warfarin was started simultaneously for anticoagulant therapy. Patients who had Loeffler endocarditis with evidence of intracardiac thrombus detected by echocardiography or CMR, and one patient taking warfarin, had bleeding. One (3.1%) patient (3.1%) [26] received heart transplantation, whereas two (6.1%) patients [15, 18] underwent surgical excision of right ventricular (RV) and LV thrombus and fibrosis and mitral valve replacement. Eight patients died from cerebral embolus [28, 34, 37] (37.5%), heart failure [31, 36] (25%), and bacterial sepsis [12, 15, 36] (37.5%). The cause of death was not clarified in one patient. Three patients [18, 26, 31] were readmitted with severe congestive heart failure. Heart failure (*P* = 0.008) and the absence of anticoagulation treatment (*P* = 0.021) were more common pessimistic outcomes (Table 3). The thrombus was completely resolved in 42.4% of patients, and no further events were reported after the hospital discharge follow-up.

**Table 3:** Comparison between those with favorable and poor outcomes (2 cases not available)

	Favorable outcomes (n = 19)	Poor outcomes (n = 12)	*p* value
Mean age	43.1	45.4	0.681^‡^
Male	3	9	0.002^§^
Heart failure	5	9	0.008^§^
Stoke	6	4	0.919^§^
Without steroid	1	2	0.296^§^
Without antithrombotic	3	7	0.021^§^

^‡^*p* value was computed by Independent samples *t*-test

^§^*p* value was computed by Fisher's exact test

## Discussion and conclusion

We described a case presenting with embolic strokes secondary to Loeffler endocarditis with intracardiac thrombus. Also, we provided data based on a series of published cases to summarize the clinical presentation, diagnostic findings, treatment, and outcomes of patients with intracardiac thrombus-proven Loeffler endocarditis. The mortality was found to be high (27.3%) in these recruited patients, and patients with heart failure and those without anticoagulation treatment were associated with poor outcomes.

Cardiac involvement was frequently reported to be related to Loeffler endocarditis in HES, which was characterized by eosinophilic myocardial infiltration and necrosis in the acute necrotic stage. This damage was followed by a chronic fibrotic stage that involved the formation of an intracardiac thrombus with a frequent preference for the apex. This eventually led to restrictive cardiomyopathy and congestive heart failure, which portended an unfavorable prognosis. Our results showed that patients who had Loeffler endocarditis with endoventricular thrombus had a wide age distribution; the disease occurred in patients aged as young as 4 years and as old as 74 years. Women were often more affected than men. Elevated serum troponin levels were found both in the case we described and in most cases we recruited. This might be due to the release of toxic cationic proteins from degranulating eosinophils or pump failure or vascular damage caused by myocardial necrosis [38]. Cardiac markers might be sensitive indicators of persistent eosinophils related to myocardial damage [39]. In addition, echocardiography can provide useful information, such as endomyocardial thickening, left and RV thrombus formation, and valvular regurgitation [40]. Of note, Loeffler endocarditis with a small thrombus in the thrombolysis stage might be difficult to diagnose using echocardiography and is sometimes confused with apical hypertrophy, such as that in our case. CMR can clearly identify apical thrombus and diffuse subendocardial fibrosis [41]. Consistent with a significant number of patients, the diagnosis of our case depended on the presence of HES in combination with cardiac involvement on CMR. Hence, if the diagnosis of Loeffler endocarditis with thrombus is under suspicion, CMR is quite informative. Endomyocardial biopsy remains the gold standard but is fraught with risks, such as sampling errors or iatrogenic embolism [42]. Furthermore, the presence of intracardiac thrombus can increase the risk of thromboembolism during an endomyocardial biopsy.

The goals for the treatment of Loeffler endocarditis are to reduce potentially eosinophil-mediated end-organ damage and prevent adverse thrombotic events. Our limited literature showed that 33.3% of patients who had Loeffler endocarditis with intracardiac thrombus developed thromboembolic complications, and the mortality rate was 27.3%. Previous studies showed that thromboembolic disorders associated with Loeffler endocarditis were particularly difficult to control, despite anticoagulation therapy with warfarin, and embolic complications still appeared. One mechanism might involve the release of eosinophilic granular proteins from eosinophils [43], which could neutralize thrombomodulin via electrostatic binding, resulting in thromboembolism. Consistent with the aforementioned findings, thrombus regression occurred in 14 patients (42.4%) after treatment with heparin or vitamin K antagonists both in our reported patient and patients included in this review. The risks of embolic events and mortality in these patients were much higher than those in patients with LVT caused by acute myocardial infarction; however, the rate of thrombus resolution in the recruited patients was relatively lower [44, 45]. The poor outcome suggests that once we identify patients with LVT in clinical practice, HES with cardiac involvement should be taken into consideration. Consistent with our case, several case series demonstrated that most patients who received steroid therapy had hematologically normalized eosinophilia, and cardiac symptoms improved significantly. However, determining the preferred therapy other than corticosteroid therapy as the initial treatment of patients was also the essential step, such as patients with known imatinib-sensitive mutations and myeloproliferative HES. Additional immunosuppressive treatment with cyclophosphamide or azathioprine, as well as other cytotoxic agents or interferon-alpha, is usually reserved for patients with corticosteroid treatment failure. In our study, although one patient received endomyocardial stripping treatment, unfortunately, hypereosinophilia relapsed after 2.5 years. Valve replacement for five patients with severe valvular regurgitation provided considerable benefits. Limited experience with valve replacement/repair and endomyocardial stripping in Loeffler’s study. In addition, consistent with other cases, our study showed that warfarin might have a clear therapeutic benefit in anticoagulation for Loeffler endocarditis with intracardiac thrombus.

Our study was limited by the small number of patients. Also, all data were derived from published cases, leading to publication bias. However, keeping in mind the rarity of Loeffler endocarditis, large-scale prospective or retrospective studies might be more difficult to conduct. Furthermore, the real mortality rate remains difficult to estimate, and cases not critical or with nonspecific symptoms are recorded at a lower rate. Therefore, mortality might be overestimated.

Loeffler endocarditis with intracardiac thrombus is rare, but the mortality is high. Our study highlighted the importance of CMR in establishing the diagnosis and monitoring treatment in Loeffler endocarditis. Early treatment with corticosteroids after excluding secondary causes without delay may be beneficial for these patients. In addition, late recurrence may occur, and long-term follow-up is required.

## Data Availability

All the data discussed in the manuscript are included in this published article.

## Abbreviations

HES: Hypereosinophilic syndrome
LV: Left ventricular
RV: Right ventricular
